# Drought mildly reduces plant dominance in a temperate prairie ecosystem across years

**DOI:** 10.1002/ece3.6400

**Published:** 2020-06-01

**Authors:** Karen Castillioni, Kevin Wilcox, Lifen Jiang, Yiqi Luo, Chang Gyo Jung, Lara Souza

**Affiliations:** ^1^ Oklahoma Biological Survey Department of Microbiology and Plant Biology University of Oklahoma Norman OK USA; ^2^ Ecosystem Science and Management University of Wyoming Laramie WY USA; ^3^ Center for Ecosystem Science and Society Northern Arizona University Flagstaff AZ USA

**Keywords:** climate change, clipping, disturbance, Drought‐Net, mixed‐grass prairie, species reordering

## Abstract

1. Shifts in dominance and species reordering can occur in response to global change. However, it is not clear how altered precipitation and disturbance regimes interact to affect species composition and dominance.
2. We explored community‐level diversity and compositional similarity responses, both across and within years, to a manipulated precipitation gradient and annual clipping in a mixed‐grass prairie in Oklahoma, USA. We imposed seven precipitation treatments (five water exclusion levels [−20%, −40%, −60%, −80%, and −100%], water addition [+50%], and control [0% change in precipitation]) year‐round from 2016 to 2018 using fixed interception shelters. These treatments were crossed with annual clipping to mimic hay harvest.
3. We found that community‐level responses were influenced by precipitation across time. For instance, plant evenness was enhanced by extreme drought treatments, while plant richness was marginally promoted under increased precipitation.
4. Clipping promoted species gain resulting in greater richness within each experimental year. Across years, clipping effects further reduced the precipitation effects on community‐level responses (richness and evenness) at both extreme drought and added precipitation treatments.
5. *Synthesis:* Our results highlight the importance of studying interactive drivers of change both within versus across time. For instance, clipping attenuated community‐level responses to a gradient in precipitation, suggesting that management could buffer community‐level responses to drought. However, precipitation effects were mild and likely to accentuate over time to produce further community change.

## Graphical abstract

Shifts in dominance and species reordering can occur in response to global change. We manipulated an experimental precipitation gradient combined with clipping in a temperate mixed‐grass prairie to examine how both treatments can shape plant communities. We found that initial shifts in abundance were detected by examining species‐ to community‐level changes over time; drought increased evenness and this was related to the decline of the dominant species and increase in subdominants, while mesic conditions mildly promoted plant richness.

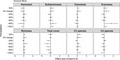

## 1 INTRODUCTION

Climatic changes are altering Earth's hydrological cycle, resulting in altered precipitation amounts, and increased frequency and magnitude of extreme wet and dry years (IPCC, 2013). These trends will likely continue into the future with plant communities expected to undergo significant changes in ecological structure (Smith, Knapp, & Collins, 2009). It is especially important to understand the magnitude of climatic impacts in grasslands due to their unique sensitivity to changes in precipitation (Huxman et al., 2004; Knapp, Briggs, & Koelliker, 2001; Sala, Gherardi, Reichmann, Jobbágy, & Peters, 2012; Wilcox et al., 2017). Altered precipitation can lead toward shifts in the distribution and abundance of plant species, impacting species composition at local scales (Sala et al., 2012). The rate by which grasslands will respond to changes in precipitation will vary among grasslands types, xeric versus mesic, and will depend on the life history of organisms (Sala et al., 2012). Thus, assessing ecological responses to multiple drivers and how they interact will allow us to document and better predict responses in a highly responsive ecosystem (Jones, Ripplinger, & Collins, 2017).

Reordering of species dominance patterns or the changes in the relative abundances of species within a community over time, and changes in species composition (e.g., colonization and local extinction) underpin important community dynamics under global change (Jones et al., 2017). Rates of response to global change may be dependent on how species are organized in a community (Smith et al., 2009). Species in a given community may be ranked by their dominance reflecting their success in competing for light, water, and nutrients. The mass ratio hypothesis postulates that dominant species use the majority of resources and have disproportionally large community impact (Grime, 1998). At intermediate resources levels, subdominants can become more abundant having greater effects on the ecosystem, but they become more important as resource levels increase or decrease with climatic perturbations (Mariotte, 2014). As a result, the responses of these species to climate change can determine the rate at which other species can respond (Felton & Smith, 2017; Smith et al., 2009).

Grassland subdominants often thrive under unstable climate conditions, including across wet and dry years (Grime, 1998). Subdominants can enhance community resistance against drought by increasing their aboveground biomass production (Mariotte, Vandenberghe, Kardol, Hagedorn, & Buttler, 2013). Dominant species are expected to respond to changes in climate most directly (Felton & Smith, 2017; Smith et al., 2009), whereas subdominant species may respond to climate change directly and indirectly through their interactions with the dominant species (Barton, Beckerman, & Schmitz, 2009; Belote, Weltzin, Norby, & Weltzin, 2009; Kardol et al., 2010). For instance, Kardol et al. (2010) showed that the proportion of subdominant species increased under dry compared with wet conditions. Further, Kardol et al. (2010) found that dominant species responded most strongly to the direct impacts of drought, while subdominant species responded to the resulting decrease in the strength of competition interactions with the dominant species. Because responses to climate change differ among individual plant species and depend on community context (Parmesan & Yohe, 2003; Tylianakis, Didham, Bascompte, & Wardle, 2008; Zavaleta et al., 2003), the resultant community dynamics are difficult to predict. Thus, assessing climate change effects on the entire community and on dominant, subdominant, and transient (i.e., species not persistent in the vegetation) community members separately is necessary (Mariotte, 2014).

In the US Great Plains, both grazing by large ungulates and hay harvesting are strong drivers of plant community structure and ecosystem functioning (Collins, Knapp, Briggs, Blair, & Steinauer, 1998; Knapp et al., 2008; Koerner & Collins, 2014; Shi et al., 2016). Both grazing and hay harvest are disturbances that remove aboveground vegetation, consequently altering species‐level plant species abundances (Borer, Seabloom, Gruner, Harpole, & Hillebrand, 2014; Shi et al., 2016), community‐level biodiversity (Collins et al., 1998), and productivity (Collins et al., 2011; Smith et al., 2009). Hay harvesting can also suppress the growth of competitive dominant species, promoting community‐level biodiversity by promoting resource availability to subdominant species (Borer et al., 2014; Collins et al., 1998; Shi et al., 2015). An understanding of how hay harvest and rainfall interact to structure plant communities is necessary not only to effectively manage these systems, but also to provide new insights into how multiple forms of disturbance interact to shape the dynamics of natural systems (Riginos, Porensky, Veblen, & Young, 2018).

Here, we assessed the effects of a manipulated precipitation gradient, and its effects concurrent with clipping (i.e., simulating vegetation disturbance) on community structure. First, we predicted that subdominant species would increase in abundance resulting from a decline in abundance of dominant species as the environment becomes drier and harsher. Similarly, transient species would increase in abundance and frequency under increased drought or increased water availability. This change in the community dynamics would be reflected in biodiversity metrics by increasing richness and evenness as subdominant and transient species thrive under altered resource availability. Second, clipping acting independently would increase subdominant and transient species by reducing the abundance of dominant species. Consequently, richness and evenness would increase promoting biodiversity. Third, clipping would enhance the effects of drought and increased water availability by reducing the abundance of dominant species and promoting transient and subdominant species.

## 2 METHODS

### 2.1 Study site

We established this field experiment in an existing temperate mixed‐grass prairie grassland at Kessler Atmospheric and Ecological Field Station (KAEFS, http://kaefs.ou.edu/), central Oklahoma, USA (34°59'N, 97°31'W). KAEFS was abandoned from field cropping in 1973 but has sustained light grazing in designated areas (Xu, Sherry, Niu, Li, & Luo, 2013). The grassland is dominated by C_4_ and C_3_ graminoids, and forbs (species list in Table S1). The mean annual precipitation from 1994 to 2018 was 885 mm, and from 1997 to 2018, the mean annual air temperature was 16.2°C (Oklahoma Climatological Survey, Norman, OK, USA). In 2017 and 2018, total rainfall was 992.1 mm and 1,241.0 mm, respectively. Mean annual air temperature for both years was 17˚C and 16˚C (Figure S1). The soil is classified as the Nash‐Lucien complex, characterized by a neutral pH, high water holding capacity (around 37%), a depth of about 70 cm, and a moderately penetrable root zone (Xu et al., 2013).

### 2.2 Experimental design

#### 2.2.1 Treatments description

In Spring 2016, we installed rain interception shelters to impose a gradient of precipitation treatments, as part of a global coordinated experimental network (Drought‐Net: http://wp.natsci.colostate.edu/droughtnet/). The experimental design consisted of seven levels of precipitation, establishing a precipitation gradient: −100%, −80%, −60%, −40%, −20% rainfall exclusion, 0% change in precipitation (i.e., control) and precipitation addition +50%, in a fully factorial randomized block design (*n* = 3, *N* = 21, Figure S2). Rain interception shelters were made of acrylic transparent plastic that blocked rain but not sunlight, and they were present in all treatments, including control, to exclude confounding effects of shelter presence (Beier et al., 2012; Yahdjian & Sala, 2002). Rain gauges were used to estimate rainfall collected by each treatment, which coincided closely with our target manipulation levels (G. Newman, “unpublished data”). We set up the +50% precipitation addition plots by adding panels on two sides of plots receiving ambient rainfall to divert additional precipitation onto the plot. The width of each additional panel sheet was 25% the width of the experimental plot, together equaling 50% of the plot (Figure S2). Precipitation collected from panels was drained by gutters to the inside of the plot. Thus, the frequency of precipitation addition and total precipitation amount coincided with the ambient precipitation events. Each 4 × 4 m experimental plot was subdivided into four 1 × 1 m subplots, with a 1 m buffer area on the edge of each plot. In addition to precipitation, one subplot was clipped at the end of the growing season in September 2016, 2017, and 2018 to remove aboveground biomass at a height of 10 cm from ground level once a year to mimic hay harvesting. Similar to hay production, clipped materials were removed from subplots (Xu et al., 2013). Diagonally from the clipping subplot was the unclipped control subplot (Figure S2).

#### 2.2.2 Soil moisture content and temperature

We measured volumetric soil water content (VWC, m^3^/m^3^) and soil temperature (˚C) every 30 min from September 2016 to September 2018 using Decagon 5TM soil probes with a depth of 1–10 cm in each clipped and unclipped subplot. During the growing season (May to September), the precipitation gradient significantly altered VWC (Table S2) in 2017 (*F* = 156.8 and *p* < .001) and in 2018 (*F* = 52.76 and *p* < .001), while soil temperature (Table S2) in 2017 (*F* = 88.4 and *p* < .001) and in 2018 (*F* = 72.74 and *p* < .001). However, we found significant effects of clipping on soil temperature only in 2018 (*F* = 16.92 and *p* < .001). We found no significant interaction between the precipitation gradient and clipping to affect VWC and soil temperature in both years (*p* > .05).

### 2.3 Plant species‐specific and community‐level responses

To examine the main and interactive effects of clipping and the gradient of precipitation on two levels of organization (i.e., species‐specific and community wide), we tallied the number of species in each subplot (richness) and estimated species‐specific foliar cover (%) twice a year in May and August. We estimated percent foliar cover (e.g., vegetative cover including stems and leaves) in the one clipped and the one unclipped subplot by using a modified Braun‐Blanquet cover‐abundance scale that included seven categories of percent foliar cover: 1%, 1%–5%, 5%–25%, 25%–50%, 50%–75%, 6:75%–95%, and 7:95%–100% (Braun‐Blanquet, 1932); we used the median of each assigned cover class as the abundance for each species in a subplot. We used maximum percent foliar cover between May and August sampling periods as species abundance values for each species in each year. Next, species‐specific relative abundance was obtained by dividing species‐specific abundance to the sum of all species abundance per plot. Relativized cover allows for comparison of species composition across years with different absolute abundance values coinciding with interannual variation in environmental characteristics (e.g., in a dry versus a wet year). Jaccard's index (evenness) was calculated using foliar cover data. We also calculated the average abundance of C_3_ and C_4_ species, subdominants and transients from relative cover data. We defined plant species as “dominant,” “subdominant,” or “transients” based on frequency of occurrence and relative species cover. Dominant plant species were considered species having relative cover of >45%, subdominant species were those with relative cover values between 0.2% and 45%, and transient species were determined as those having <0.2% relative abundance.

### 2.4 Data analysis

#### 2.4.1 Species and community shifts through time

To assess for directional changes in species and community‐level trajectory in reference to baseline measurements (i.e., prior treatment application in year 2016), we computed Cohen's d effect size (Cohen, 1988), that is, the standardized mean difference using the pooled standard deviation of the treatment and control groups with a bias correction (Hedges & Olkin, 1985). Specifically, treatment and control plots in 2018 were compared with their 2016 pretreatment data. This allows for comparison of species and community shifts occurring in the background community with shifts occurring due to treatments. Effect size was calculated using function cohen.d in the effect size package in R (Torchiano, 2019). Data visualization was created by using ggplot2 (Wickham, 2016).

#### 2.4.2 Precipitation gradient and clipping effects

To determine species and community‐level responses to treatments within each year, we used generalized linear models with mixed‐effects models and ANCOVA. We assessed differences among clipping and precipitation treatments for individual species covers, total subdominant species cover, total transient species cover, total C_3_ species cover, total C_4_ species cover, species richness, and species evenness using the glmer function in the lmerTest package (Bates, Mächler, Bolker, & Walker, 2015) and ANOVA function in the car package in R (Fox & Weisberg, 2019). We ran a single model separately for 2017 and 2018 having precipitation, clipping, and precipitation*clipping (i.e., 2017 and 2018) as main fixed effects, while block and plot as random factors in the glmer model. We treated both block and plot as random factors in the model to account for uncontrolled variation among blocks and plots. The level of significance for all statistical tests was α = 0.05. Choice of error distribution was dictated by the scale of the response variable. A Poisson distribution and log link were chosen to model richness as a count variable. Evenness and total absolute cover were modeled with a gamma distribution log link and inverse link, respectively, as they have only nonnegative values. All relative cover variables were modeled with binomial distribution with logit link and weighted by total absolute cover. Tests of fixed effects were obtained with Type II Wald chi‐square tests.

#### 2.4.3 Species gains, losses, and turnover

We applied RAC_change() function (Avolio et al., 2019) in codyn package to calculate species gain and loss within each plot from 2017 to 2018. Species gains and losses were then compared across precipitation and clipping treatments using ANCOVA.

#### 2.4.4 Species composition

We used nonparametric, permutational multivariate analysis of variance (PERMANOVA) to determine the difference among communities across precipitation and clipping treatments, which were treated as fixed factors in the model. We performed the PERMANOVA on a Bray–Curtis similarity matrix generated from the log‐transformed (log X + 1) plant composition data (i.e., species‐specific relative percent foliar cover). We followed up PERMANOVA analyses with permutational multivariate analysis of dispersion (PERMDISP) to assess heterogeneity of local communities within treatments (Anderson, 2001). Plant compositional analyses were conducted using package vegan (Oksanen et al., 2019).

## 3 RESULTS

### 3.1 Precipitation gradient effects

#### 3.1.1 Species and community shifts across time

**Figure 1:**
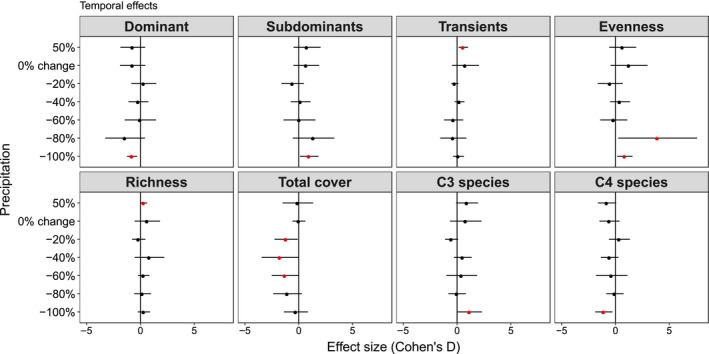
Average effect sizes (Cohen's D) and 95% confidence interval (bars) of relative abundance (foliar cover %) for community‐level within precipitation treatments. Note that evenness is based on Jaccard index and richness is based on the number of species. Year 2018 (after treatment application) was compared to year 2016 (before treatment application), including the control treatment (i.e. 0% change in precipitation). Red circles + bars denote significant effect sizes; red circles + bars to the right indicate positive effect sizes; and red circles + bars to the left indicate negative effect sizes

Extreme drought had a positive effect on evenness (Cohen's d of 0.71 standard deviations (SD)) while added precipitation had a positive effect on richness (0.31SD, Figure 1). Greater plant evenness in drier treatments was concurrent with reduced abundance of the dominant species and C_4_ species in our system (*Schizachyrium scoparium*, referred to as dominant species hereafter: −0.47SD) and increased subdominant species abundance (0.80SD, Figure 1). Two C_3_ forbs species (*Ambrosia psilostachya*: 1.00SD, and *Dalea purpurea*: 0.38SD) and a C_4_ grass species (*Sorghastrum nutans*: 0.95SD) increased greatly in droughted plots. In contrast, increased richness in 50% precipitation addition occurred concurrently with increased abundance of transient species (0.43SD, Figure 1). Total absolute cover was lower in −60% (−1.24 SD), −40% (−1.70SD), and −20% (−1.25SD) precipitation reduction.

#### 3.1.2 Within year precipitation effects

Neither relative abundances of groups (dominant, subdominant, transient, C_3_, C_4_) nor richness or evenness were influenced by precipitation treatments within any year (Table 1, Tables S5 and S6). However, we found increased dissimilarity of species composition under + 50% precipitation compared to 0% change in precipitation (Table S3). Dispersion within “added precipitation” treatments (e.g., increased dissimilarity) did not coincide with species gains (*F* = 1.19 and *p* = .28), losses (*F* = 1.39 and *p* = .25), or species turnover (*F* = 0.00 and *p* = .92) (Table S7).To further explore increased dissimilarity patterns, we subsequently generated ranked abundance curves for each experimental replicate in each precipitation level (Figure S6). Rank abundance curves illustrate how shifts in plant dominance across replicates contribute toward variability in species composition in precipitation extremes.

**Table 1:** Model summary and ANCOVA results for generalized linear models of main and interactive effects of precipitation and clipping on community‐level and species‐specific responses

Community‐level	Precipitation		Clipping		Precip. *x* Clip.	
	Chisq	*P*	Chisq	*P*	Chisq	*P*
Richness (S)						
2017	2.06	.15	4.12	**.04**	0.19	.67
2018	0.94	.33	35.85	**.00**	1.03	.31
Evenness (J’)						
2017	0.40	.52	1.48	.22	2.28	.13
2018	0.15	.70	17.45	**.00**	0.09	.76
Dominant						
2017	0.46	.50	1.87	.17	20.98	**.00**
2018	1.69	.19	26.74	**.00**	3.18	.07
Subdominants						
2017	0.55	.46	0.44	.51	19.10	**.00**
2018	1.83	.18	27.03	**.00**	2.22	.14
Transients						
2017	0.00	.95	9.57	**.00**	3.30	.07
2018	0.58	.45	36.02	**.00**	7.65	**.01**
C3 species						
2017	0.50	.48	5.60	**.02**	16.59	**.00**
2018	0.27	.60	2.97	.08	0.49	.48
C4 species						
2017	0.59	.44	2.12	.15	17.15	**.00**
2018	0.15	.69	3.32	.07	0.09	.76
Total absolute cover						
2017	0.07	.78	0.06	.80	0.25	.61
2018	1.32	.25	1.68	.19	0.06	.81
Species‐specific: forbs						
*Ambrosia psilostachya*						
2017	0.13	.72	0.02	.87	14.79	**.00**
2018	1.32	.25	11.37	**.00**	2.41	.12
*Calylophus serrulatus*						
2017	0.51	.48	22.48	**.00**	0.42	.52
2018	0.90	.34	16.28	**.00**	0.09	.76
*Croton monanthogynus*						
2017	5.59	**.02**	31.69	**.00**	12.59	**.00**
2018	3.07	.08	3.74	.05	0.27	.61
*Dalea purpurea*						
2017	0.13	.72	6.41	**.01**	0.29	.59
2018	0.16	.69	1.48	.22	11.04	**.00**
*Erigeron strigosus*						
2017	7.25	**.01**	47.05	**.00**	2.85	**.09**
2018	2.53	**.11**	22.79	**.00**	12.34	**.00**
*Lespedeza cuneata*						
2017	0.17	.68	30.43	**.00**	1.07	.30
2018	0.15	.70	3.17	.07	16.62	**.00**
*Solidago rigida*						
2017	0.21	.64	0.34	.56	0.35	.55
2018	1.36	.24	6.28	**.01**	38.26	**.00**
*Symphyotrichum ericoides*						
2017	0.33	.56	0.01	.92	0.64	.42
2018	0.50	.48	54.97	**.00**	0.02	.89
Species‐specific: graminoids						
*Bothriochloa ischaemum*						
2017	2.37	.12	0.78	.38	3.45	.06
2018	0.61	.43	5.68	**.02**	19.06	**.00**
*Dichanthelium oligosanthes*						
2017	0.49	.48	13.72	**.00**	5.12	**.02**
2018	0.78	.38	25.27	**.00**	1.08	.30
*Sorghastrum nutans*						
2017	3.09	.08	14.04	**.00**	0.30	.58
2018	0.06	.80	8.85	**.00**	25.62	**.00**
*Sporobolus compositus*						
2017	0.00	.95	10.19	**.00**	0.01	.93
2018	0.28	.60	80.65	**.00**	0.01	.92

Note

Precipitation (covariate), clipping, and their interaction were treated as main fixed factors, with block and plot as random factors. Significant results (*p* < .05) are shown bold. Overall *df *= 1.

### 3.2 Clipping effects

#### 3.2.1 Species and community shifts across time

**Figure 2:**
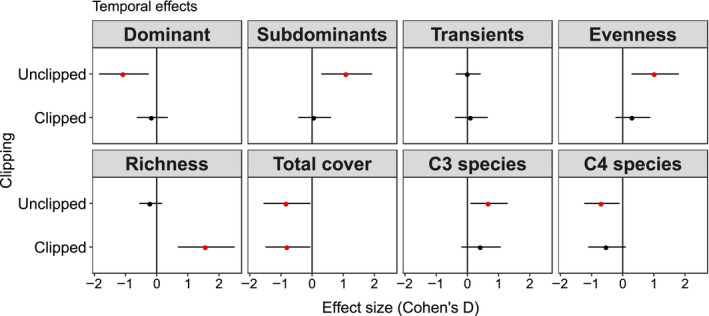
Average effect sizes (Cohen's D) and 95% confidence interval (bars) of relative abundance (foliar cover %) for community‐level in clipped versus. unclipped condition. Note that evenness is based on Jaccard index, and richness is based on the number of species. Year 2018 (after treatment application) was compared to year 2016 (before treatment application), including the control treatment (i.e. unclipped plots). Red circles + bars denote significant effect sizes; red circles + bars to the right indicate positive effect sizes; and red circles + bars to the left indicate negative effect sizes

Clipping had a positive effect on richness from 2016 to 2018 (Cohen's d of 0.68 SD), while in unclipped plots we observed an increase in evenness (0.76 SD), subdominant abundance (0.18 SD), and overall C_3_ species abundance (0.60 SD, Figure 2) over time. Unclipped plots, however, experienced reduced abundance of C_4_ species (−0.60SD, Figure 2) and dominant species over time (−0.49SD, Figure 2). Total abundance cover was negatively influenced independently of the treatment (Figure S4). Across time, clipped plots gained 87% more species compared to unclipped (*F* = 32.7 and *p* < .001), while unclipped plots lost 28% species (*F* = 13.24 and *p* < .001) (Table S7).

#### 3.2.2 Within year clipping effects

Clipping effects generally promoted richness while minimally altering plant dominance. In 2018, richness was on average 24 species in clipped and 15 species in unclipped conditions, while evenness was on average 0.71 in clipped compared to 0.77 in unclipped plots. Evenness values were not significantly different between clipped and unclipped treatments in 2017 (Table 1, Tables S5). Subdominants significantly decreased in clipped plots (2018: *F* = 27.03 and *p* < .001, 0.66% average relative abundance), compared to unclipped plots (0.72% average relative abundance). Alternately, transients increased in clipped plots (0.20% average relative abundance, *p* < .001) compared to unclipped plots (0.15% average relative abundance) in both years (Table 1). Total absolute cover remained unchanged (*p* > .05).

Species compositional similarity was significantly different between clipped and unclipped plots based on PERMANOVA in 2018 (Table S3), meaning that species composition was more different in clipped versus unclipped treatments.

### 3.3 Clipping‐precipitation interaction

#### 3.3.1 Species and community shifts across time

**Figure 3:**
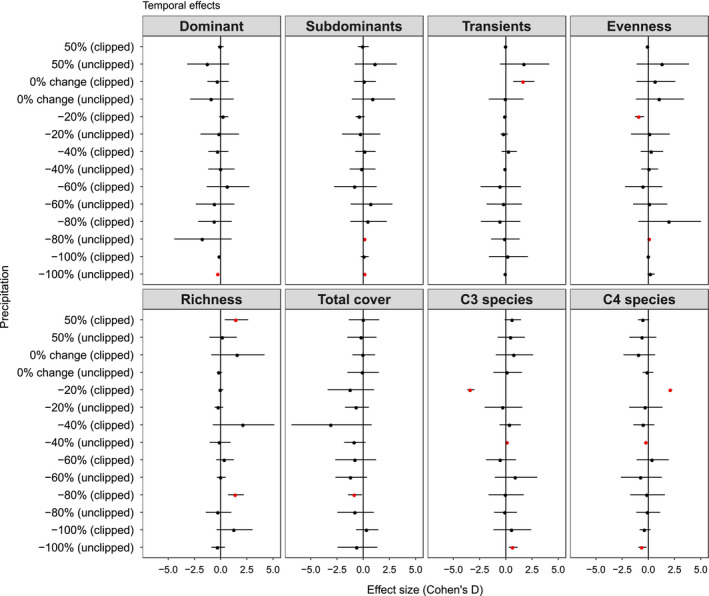
Average effect sizes (Cohen's D) and 95% confidence interval (bars) of relative abundance (foliar cover %) for community‐level within precipitation treatments in clipped versus. unclipped condition. Note that evenness is based on Jaccard index and richness is based on number of species. Year 2018 (after treatment application) was compared to year 2016 (before treatment application), including the controls treatments (i.e., 0% change in precipitation and unclipped plots). Red circles + bars denote significant effect sizes; red circles + bars to the right indicate positive effect sizes; and red circles + bars to the left indicate negative effect sizes

Interactive effects between precipitation and clipping were minimal, despite a few differences. Clipping had a positive effect on richness not only when we added 50% precipitation (1.11 SD), but also when we reduced precipitation by 80% (0.75 SD, Figure 3).

#### 3.3.2 Within year precipitation x clipping effects

Interactive effects of precipitation x clipping were more consistent at the species‐ than community‐level (Table 1). For example, the subdominant grass *S. nutans* was slightly more abundant in most of the precipitation reduction plots, in clipped conditions (average 0.05% relative abundance) compared to unclipped (average 0.04% relative abundance) (Table 1). In contrast, added precipitation had a negative effect on *S. nutans*, which declined in clipped plots (0.04% relative abundance) relative to unclipped plots (0.07% relative cover). Additionally, *A. psilostachya* and *Croton monanthogynus,* both herbaceous forbs*,* increased in foliar cover with drought in clipped relative to unclipped conditions, while *E. strigosus* showed the opposite pattern. On the other hand, *Solidago rigida,* another herbaceous forb*,* became more abundant from 0.002% relative abundance in unclipped plots to 0.10% relative abundance in clipped plots with water addition. Our analysis revealed no significant interaction of precipitation and clipping to have no influence on species composition (Table S3), other plant community metrics (Table S4), changes in species gains (*F* = 1.75 and *p* = .19), losses (*F* = 0.05 and *p* = .82), and plant species turnover (*F* = 0.71 and *p* = .40). However, changes occurred in species composition at the plot level (within replicates) in the first year only (Table S3). Finally, we did find an interactive effect on transient species abundance in 2018 (*F* = 7.65 and *p* = .01). In that year, clipping increased transient relative abundance from 0.23% relative abundance in clipped ambient plots to 0.27% relative abundance in + 50% precipitation plots.

## 4 DISCUSSION

### 4.1 Biodiversity change occurs across years

Our study demonstrated that initial shifts in abundance were detected by examining species‐ to community‐level changes over time. Across years, in dry conditions we documented an increase in evenness that was related to the decline of the dominant species and increase in subdominants, while mesic conditions mildly promoted plant richness. Clipping enhanced plant richness not only over time through species gains, but also in each year. When combining altered precipitation with clipping, specifically under mild drought, we observed a decline in evenness that was related to the reduced abundance of C_3_ species and increase in C_4_ species. However, in extreme dry levels, clipping muted the effects on the dominant plant species, plant evenness, functional groups (C_3_ and C_4_ species), and subdominants.

As current climate change predictions for the Great Plains point to increased frequency and duration of severe droughts, these short‐term results suggest the first signals of species shifting dominance patterns. Plant species seem to be tracking environmental conditions through reducing or increasing their abundance within the existing community. Detecting changes that occur in the short‐term may predict abrupt reshuffling of plant communities which could ultimately lead to the formation of novel species assemblages (Walther, 2010).

### 4.2 Precipitation gradient

We predicted that subdominant species, including C_3_ species, would become more abundant to the detriment of dominant species as the environment became drier. As predicted, we found that extreme drought conditions decreased the dominant species abundance, while we observed an increase of subdominants and C_3_ species over time. These results refer to across time analysis since no within‐year effects of precipitation were detected. Similarly, Mariotte et al. (2013) found evidence for subordinate species increase enhancing their aboveground biomass production under drought, with decreased competitiveness of dominant species. Mariotte (2014) further suggests that subordinate plant species may have larger impacts on ecosystem functioning than expected and more experiments should study the role of subordinate species under present and projected climate.

Shifts in species‐specific abundance escalated to changes in plant evenness in extreme drought by shifting plant dominance patterns. In contrast, added precipitation had a marginal positive impact on plant richness, particularly by promoting the abundance of transient, but not affecting subdominant species. Similarly, subdominant species were previously found not to be influenced by added precipitation in a mixed‐grass prairie (Zelikova et al., 2014). Interestingly, these findings were only notable when taking into account initial variation in plant distribution and abundance (Langley et al., 2018).

Although at the community‐level, we documented increased dissimilarity in precipitation extremes across replicates in 2017 and 2018, composition has not fully changed for all plots. This is likely because some plots might be changing at a faster pace than others. We speculate that as species try to adapt to extreme changes in resources, their abundance may shift and increase dissimilarity among plots of a treatment. Eventually, all the plots in a treatment may become different than the other if water availability conditions remain the same (Komatsu et al., 2019).

Various studies have reported well‐adaptation of *S. scoparium* to drought conditions (Maricle & Adler, 2011; Maricle, Caudle, & Adler, 2015). Yet, in agreement with our study, the dominant species *S. scoparium* also responded negatively to other climatic changes (warming) in the same system, while the other C_4_ grass *Sorghastrum nutans* was generally more abundant in the warmed plots (Shi et al., 2015). According to Gherardi and Sala (2015), grasses can reduce their abundance and their ability to absorb water under drought. Grasses have relatively shallow roots and use soil water located in upper layers of the soil (Nippert & Knapp, 2007). However, in our within year analysis drought positively influenced *S. nutans* growth, even though this species was found to be more sensitive to water stress in tallgrass prairie (Hoover, Knapp, & Smith, 2014; Swemmer, Knapp, & Smith, 2006). Thus, likely reduced competitive pressure with the dominant species was key to promote *S. nutans* that generally has lower dominance.

In fact, forbs that responded positively to drought over time, such as *Ambrosia psilostachya*, might have been alleviated from competitive pressure for water resources, and its deeply rooted system (Hake, Powell, McPherson, Claypool, & Dunn, 1984) likely gave this species growth advantage. Further, C_3_ species show niche differentiation in water use strategies to avoid competition with C_4_ grasses for water (Nippert & Knapp, 2007). Climatic changes such as altered precipitation and warming can drive rapid changes in plant communities, especially in herbaceous plants, many with short‐term population cycles (Gottfried et al., 2012; Kelly & Goulden, 2008). Thus, our results highlight the need to understand the species‐specific sensitivity to precipitation changes along with the influence of biotic interactions because predicted changes may vary across precipitation levels (Byrne, Adler, & Lauenroth, 2017; Tomiolo, Van Der Putten, Tielborger, & Allison, 2015).

### 4.3 Clipping alone effects

We predicted that clipping would promote the abundance of subdominant and transients by reducing the abundance of dominant species, and that as a consequence, richness and evenness would increase more in clipped than unclipped plots. Increase in richness was consistent for across and within time analyses. However, within year analysis contradicted our predictions by showing that clipping actually lowered the abundance of subdominant species allowing transients to become more abundant. This observation is likely due to higher ground surface light allowed by clipping allowing transient species to better colonize under such conditions. Evenness remained unchanged at the end of two years, showing more resistance to change as previously noted (White, Bork, & Cahill, 2014).

Within year, analysis also showed changes in species composition. Early shifts in community composition due to clipping have been widely documented (Shi et al., 2015; Teyssonneyre, Picon‐Cochard, Falcimagne, & Soussana, 2002; Yang et al., 2011). Further, our temporal analysis showed that lack of clipping was detrimental to the dominant species. Although the dominant species was not affected by clipping in our across years analysis, Shi et al. (2015) found that clipping favors this species abundance when studying sensitivity of community structure and composition in the same system. This finding indicates the importance of clipping alone for the dominant species maintenance, especially due to its grazing tolerance (proxy to clipping) and for evolving to be part of grazed systems. These responses include the maintenance of a large reserve population of buds or meristems for recovery, including maintenance of high tiller natality rates (N’Guessan & Hartnett, 2011). Annual hay harvesting is common in natural and managed ecosystems across the world, being a widely used practice in grasslands. Elucidating the effects of disturbances (Smith et al., 2009), such as biomass harvesting, will help conserve biodiversity, function, and stability of ecosystems (Yang et al., 2012).

### 4.4 Interactive effects of precipitation gradient and clipping

Our results suggest that precipitation reduction acted differently when clipping was incorporated, especially under extreme drought. This finding contrasts with our predictions of overall plant decrease by combining two stressors. Less water availability and clipping allowed the dominant species to remain unchanged over time, and other groups remained constant in −100% precipitation. Multiple environmental drivers tested in grassland, such as reduced precipitation and clipping, suggest that intermediary environmental and biological variables can ultimately directly and indirectly influence unresponsive variables (White et al., 2014). These factors could be additional factors not considered in this study, such as plant traits (Díaz et al., 2007) or plant interactions (Filazzola, Liczner, Westphal, & Lortie, 2018). Similarly to White et al. (2014) study, we also found evenness to be unresponsive with precipitation reduction. Here, we suspect that this happens because the dominant species can better thrive when all species are clipped, including the ones with more drought tolerance.

In long‐term experiments of other grassland communities, the effects of rainfall on plant composition varied in direction across herbivore treatments (Riginos et al., 2018). In Riginos et al. (2018), much of the community change in lightly grazed treatments (especially after droughts) was due to substantial increases in cover of the perennial grasses, which is comparable yet for our short‐term treatments. Therefore, clipping under extreme drought should be considered with caution given our short‐term results. Most shifts in community structure and species composition are not rapid (i.e., in 2–5 years) but can emerge over a longer term (i.e., ≥10 years) (Kroël‐Dulay et al., 2015; Shi et al., 2015, 2018), and will depend on the experimental manipulation length and number of factors manipulated (Komatsu et al., 2019).

## 5 CONCLUSION

Our results revealed that precipitation altered species and community‐level changes over time by affecting shifts in species dominance patterns (more specifically with drought reducing plant dominance). Clipping promoted richness, more than dominance patterns, leading to an increase in the number of species mostly due to greater colonization/recruitment of transient species. These short‐term findings should be taken with caution given the duration of our experiment and minimal within‐year effects, but they could be the first sign of species reordering in abundance of species within a community.

## CONFLICT OF INTEREST

The authors declare that there is no conflict of interest.

## AUTHOR’S CONTRIBUTIONS

**Karen Castillioni:** Conceptualization (lead), Data curation (lead), Formal analysis (lead), Investigation (lead), Methodology (equal), Project administration (lead), Resources (equal), Validation (lead), Visualization (lead), Writing‐original draft (lead), Writing‐review & editing (lead). **Kevin Wilcox:** Conceptualization (lead), Data curation (equal), Formal analysis (equal), Investigation (equal), Methodology (equal), Project administration (equal), Supervision (supporting), Writing‐original draft (supporting), Writing‐review & editing (supporting). **Lifen Jiang:** Conceptualization (equal), Data curation (equal), Writing‐original draft (equal), Writing‐review & editing (equal). **Yiqi Luo:** Conceptualization (equal), Data curation (equal), Funding acquisition (lead), Writing‐original draft (supporting), Writing‐review & editing (supporting). **Chang Gyo Jung:** Data curation (equal), Methodology (equal), Writing‐original draft (equal), Writing‐review & editing (equal). **Lara Souza:** Conceptualization (lead), Data curation (lead), Formal analysis (equal), Funding acquisition (equal), Investigation (lead), Methodology (equal), Supervision (lead), Validation (lead), Visualization (lead), Writing‐original draft (lead), Writing‐review & editing (lead).

## Supporting information

Supplementary Material

## Data Availability

Data available in Dryad https://doi.org/10.5061/dryad.sj3tx9629.
