# Supplementary material for: Drought mildly reduces plant dominance in a temperate prairie ecosystem across years
Source: Ecol Evol. 2020 Jun 1;10(13):6702–13. doi: 10.1002/ece3.6400 (PMC7381580; doi:10.1002/ece3.6400)
Supplement: Supplementary file 1 — Supplementary Material [file ECE3-10-6702-s001.zip › ece36400-sup-0001-Supinfo/cohen_d_community_precipitation.pdf]

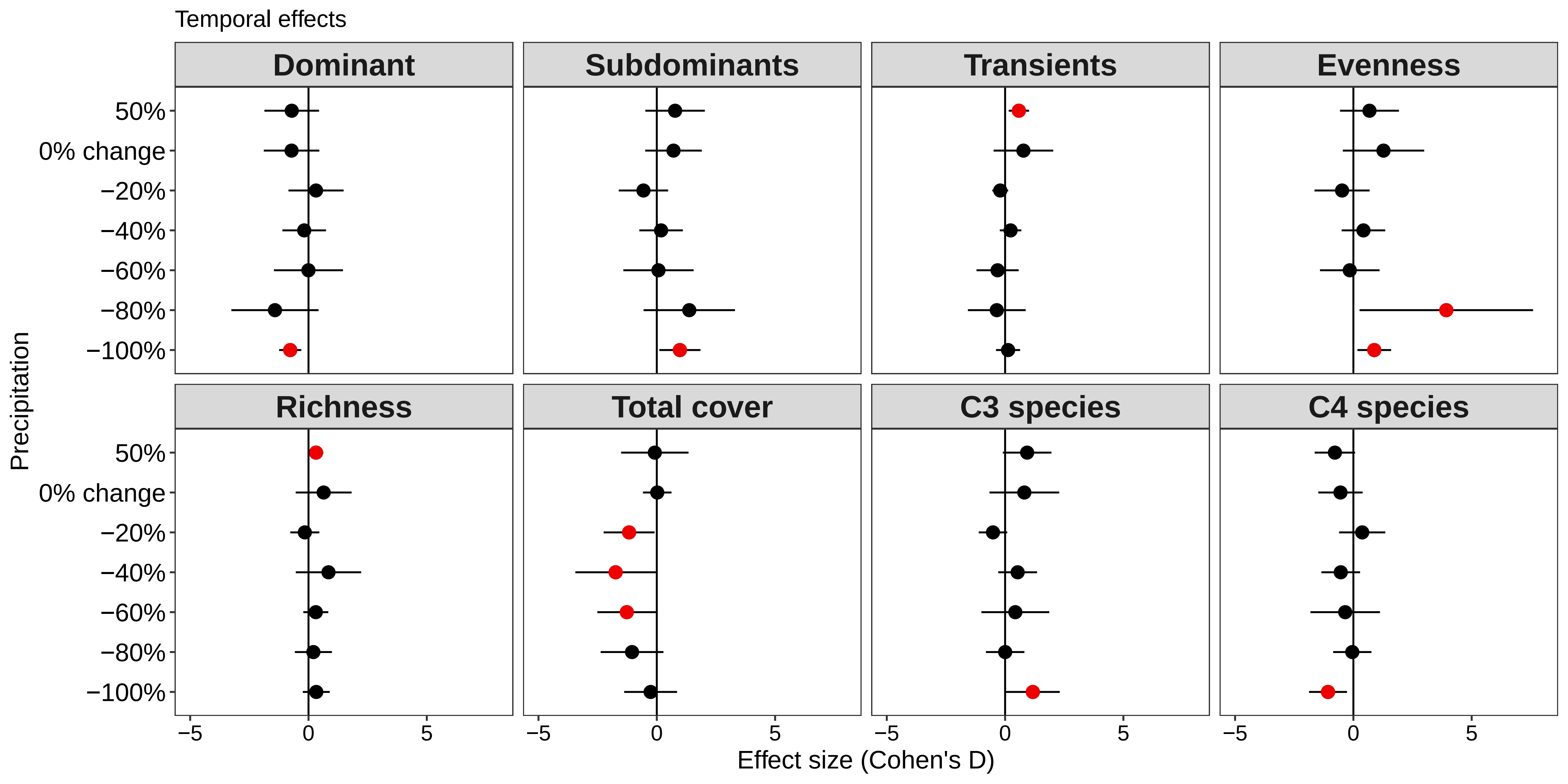
Temporal effects
Dominant
Subdominants
Transients
Evenness
Richness
Total cover
C3 species
C4 species
50%
0% change
−20%
−40%
−60%
−80%
−100%
Precipitation
−5
0
5
Effect size (Cohen's D)
