# Supplementary material for: Drought mildly reduces plant dominance in a temperate prairie ecosystem across years
Source: Ecol Evol. 2020 Jun 1;10(13):6702–13. doi: 10.1002/ece3.6400 (PMC7381580; doi:10.1002/ece3.6400)
Supplement: Supplementary file 1 — Supplementary Material [file ECE3-10-6702-s001.zip › ece36400-sup-0001-Supinfo/SupportingInformation_Castillioni_clean_version_updated.docx]

**Table 1S.** Species composition, family, functional group, growth form, life history, average cover prior treatments application in May 2016 at the Kessler Atmospheric Ecological Field Station, Washington, Oklahoma. F = family, FG = functional group, GF = growth form, LH = life history and AC = average absolute cover (%).

| **Plant species** | **F** | **FG** | **GF** | **LH** | **AC (%)** |
| --- | --- | --- | --- | --- | --- |
| *Achillea millefolium* | Asteraceae | C3 | forb | perennial | 0.39 |
| *Ambrosia psilostachya* | Asteraceae | C3 | forb | annual/ perennial | 11.61 |
| *Anemone caroliniana* | Ranunculaceae | C3 | forb | perennial | 0.02 |
| *Asclepias arenaria* | Asclepiadaceae | C3 | forb | perennial | 0.36 |
| *Asclepias stenopyllum* | Asclepiadaceae | C3 | forb | annual/ perennial | 0.37 |
| *Asclepias viridis* | Asclepiadaceae | C3 | forb | perennial | 0.39 |
| *Bothriochloa ischaemum* | Poaceae | C4 | graminoid | perennial | 3.96 |
| *Bouteloua sp.* | Poaceae | C4 | graminoid | perennial | 2.20 |
| *Calylophus serrulatus* | Onagraceae | C3 | forb | perennial | 8.08 |
| *Carex sp.* | Cyperaceae | C3 | graminoid | perennial | 0.04 |
| *Castilleja sp.* | Scrophulariaceae | C3 | forb | perennial | 0.01 |
| *Chamaecrista fasciculata* | Fabaceae | C3 | forb | annual | 0.86 |
| *Cirsium undulatum* | Asteraceae | C3 | forb | biennial/ perennial | 0.37 |
| *Coreopsis grandiflora* | Asteraceae | C3 | forb | perennial | 1.54 |
| *Croton monanthogynus* | Euphorbiaceae | C3 | forb | annual | 4.49 |
| *Dalea purpurea* | Fabaceae | C3 | forb | perennial | 1.96 |
| *Desmanthus illinoensis* | Fabaceae | C3 | forb | perennial | 0.42 |
| *Dichanthelium oligosanthes* | Poaceae | C3 | graminoid | perennial | 7.85 |
| *Eleocharis spp.* | Cyperaceae | C4 | graminoid | annual/ perennial | 0.90 |
| *Eragrostis sp.* | Poaceae | C4 | graminoid | perennial | 1.80 |
| *Erigeron strigosus* | Asteraceae | C3 | forb | annual/ biennial/ perennial | 4.5 |
| *Evax prolifera* | Asteraceae | C3 | forb | annual | 1.29 |
| *Fimbristylis puberula* | Cyperaceae | C3 | graminoid | perennial | 1.11 |
| *Galium virgatum* | Rubiaceae | C3 | forb | annual | 0.96 |
| *Hedeoma hispida* | Lamiaceae | C3 | forb | annual | 0.89 |
| *Juncus sp.* | Juncaceae | C3 | graminoid | perennial | 0.36 |
| *Juniperus virginiana* | Cupressaceae | C3 | woody | perennial | 0.02 |
| *Koeleria macrantha* | Poaceae | C3 | graminoid | perennial | 0.38 |
| *Lespedeza cuneata* | Fabaceae | C3 | forb | perennial | 2.88 |
| *Lespedeza hirta* | Fabaceae | C3 | forb | perennial | 0.75 |
| *Lespedeza sp.* | Fabaceae | C3 | forb | perennial | 0.36 |
| *Liatris squarrosa* | Asteraceae | C3 | forb | perennial | 2.23 |
| *Oxalis stricta* | Oxalidaceae | C3 | forb | perennial | 0.13 |
| *Panicum capillare* | Poaceae | C4 | graminoid | annual | 0.37 |
| *Plantago patagonica* | Plantaginaceae | C3 | forb | annual | 0.85 |
| *Plantago sp.* | Plantaginaceae | C3 | forb | annual | 0.57 |
| *Plantago virginica* | Plantaginaceae | C3 | forb | annual | 0.39 |
| *Prunus angustifolia* | Rosaceae | C3 | woody | perennial | 0.36 |
| *Psoralidium tenuiflorum* | Fabaceae | C3 | forb | perennial | 0.74 |
| *Pyrrhopappus sp.* | Asteraceae | C3 | forb | perennial | 0.04 |
| *Ratibida columnifera* | Asteraceae | C3 | forb | perennial | 0.07 |
| *Rhus copallinum* | Anacardiaceae | C3 | woody | perennial | 0.36 |
| *Rhus glabra* | Anacardiaceae | C3 | woody | perennial | 0.71 |
| *Schizachyrium scoparium* | Poaceae | C4 | graminoid | perennial | 45.89 |
| *Sisyrinchium angustifolium* | Iridaceae | C3 | forb | perennial | 0.43 |
| *Solidago ludoviciana* | Asteraceae | C3 | forb | perennial | 0.02 |
| *Solidago sp.* | Asteraceae | C3 | forb | perennial | 0.36 |
| *Sorghastrum nutans* | Poaceae | C4 | graminoid | perennial | 7.50 |
| *Sporobolus compositus* | Poaceae | C4 | graminoid | perennial | 6.62 |
| *Stenaria nigricans* | Rubiaceae | C3 | forb | perennial | 0.74 |
| *Stenosiphon linifolius* | Onagraceae | C3 | forb | perennial | 0.01 |
| *Symphyotrichum ericoides* | Asteraceae | C3 | forb | perennial | 6.31 |
| *Tetraneuris scaposa* | Asteraceae | C3 | forb | perennial | 0.77 |
| *Tragia sp.* | Euphorbiaceae | C3 | forb | perennial | 0.01 |
| *Triodanis perfoliata* | Campanulaceae | C3 | forb | annual | 0.01 |
| *Tridens flavus* | Poaceae | C4 | graminoid | perennial | 0.37 |
| *Ulmus rubra* | Ulmaceae | C3 | woody | perennial | 0.13 |
| *Verbena halei* | Verbenaceae | C3 | forb | perennial | 0.37 |

**Table 2S**. Volumetric soil water content (mean ± se, m^3^.m^-3^) and soil temperature (mean ± se, ˚C) during growing season of 2017 and 2018 across precipitation levels and clipping treatments.

|  | 2017 | 2018 |
| --- | --- | --- |
| Treatment | Volumetric soil water content (m^3^.m^-3^) | |
| Precipitation |  |  |
| -100% | 0.14 ± 0.00 | 0.13 ± 0.00 |
| -20% | 0.16 ± 0.00 | 0.14 ± 0.00 |
| -40% | 0.15 ± 0.00 | 0.14 ± 0.00 |
| -60% | 0.16 ± 0.00 | 0.15 ± 0.00 |
| -80% | 0.16 ± 0.00 | 0.16 ± 0.00 |
| 0% change (control) | 0.15 ± 0.00 | 0.15 ± 0.00 |
| 50% | 0.20 ± 0.00 | 0.17 ± 0.00 |
| Clipping |  |  |
| clipped | 0.16 ± 0.00 | 0.15 ± 0.00 |
| unclipped | 0.16 ± 0.00 | 0.15 ± 0.00 |
|  | Soil temperature (˚C) | |
| Precipitation |  | |
| -100% | 27.93 ± 0.15 | 28.90 ± 0.13 |
| -20% | 26.93 ± 0.15 | 28.10 ± 0.14 |
| -40% | 27.48 ± 0.138 | 28.52 ± 0.12 |
| -60% | 26.72 ± 0.128 | 27.97 ± 0.13 |
| -80% | 27.23 ± 0.145 | 27.82 ± 0.11 |
| 0% change (control) | 25.46 ± 0.125 | 27.16 ± 0.11 |
| 50% | 26.54 ± 0.123 | 27.41 ± 0.09 |
| Clipping |  |  |
| clipped | 26.90 ± 0.07 | 28.19 ± 0.07 |
| unclipped | 26.90 ± 0.07 | 27.82 ± 0.06 |

**Table 3S.** PERMANOVA and test for homogeneity of multivariate dispersions (PERMDISP) based on a Bray–Curtis similarity matrix of log(x+1) transformed plant species abundance. Permutations = 999. Significant results (*P* (perm) < 0.05) are shown bold; d.f. shown in parentheses.

|  | **Precipitation** | | **Clipping** | | **Precip. *x* Clip.** | | **Soil moisture** | |
| --- | --- | --- | --- | --- | --- | --- | --- | --- |
|  | F | *P* | F | *P* | F | *P* | F | *P* |
| **Species composition** |  |  |  |  |  |  |  |  |
| PERMANOVA |  |  |  |  |  |  |  |  |
| 2017 | 1.25 (1) | 0.25 | 1.05 (1) | 0.39 | 0.67 (1) | 0.75 | 0.72(1) | 0.71 |
| 2018 | 1.66 (1) | 0.07 | 3.40 (1) | **<0.01** | 0.77 (1) | 0.68 | 1.19(1) | 0.28 |
| PERMDISP |  |  |  |  |  |  |  |  |
| 2017 | 2.61 (6) | **0.03** | 0.08 (1) | 0.80 | 2.17 (13) | **0.04** | - | - |
| 2018 | 3.56 (6) | **0.01** | 0.16 (1) | 0.70 | 1.56 (13) | 0.16 | - | - |

**Table 4S.** Pairwise comparisons following PERMDISP test results on precipitation effects based on table 1. Significant results (*P* (perm) < 0.05) are shown bold.

|  | 2017 | 2018 |
| --- | --- | --- |
| Pairwise comparison | *P* | *P* |
| *-100%* precip. vs. *-80%* precip. | 0.31 | 0.31 |
| *-100%* precip. vs. *-60%* precip. | 0.30 | 0.31 |
| *-100%* precip. vs. *-40%* precip. | **0.04** | **0.01** |
| *-100%* precip. vs. *-20%* precip. | 0.84 | 0.94 |
| *-100%* precip. vs. *0%* change in precip. | 0.29 | 0.06 |
| *-100%* precip. vs. *50%* precip. | 0.79 | 0.21 |
| *-80%* precip. vs. -*60%* precip. | 0.89 | 0.87 |
| -80% precip. vs. *-40%* precip. | 0.06 | 0.16 |
| *-80%* precip. vs. *-20%* precip. | 0.06 | 0.33 |
| *-80%* precip. vs. *0%* change in precip. | 0.90 | 0.64 |
| *-80%* precip. vs. *50%* precip. | 0.16 | **0.04** |
| *-60%* precip. vs. *-40%* precip. | **0.01** | 0.09 |
| *-60%* precip. vs. *-20%* precip. | **0.05** | 0.38 |
| *-60%* precip. vs. *0%* change in precip.. | 0.82 | 0.43 |
| *-60%* precip. vs. *50%* precip. | 0.14 | **0.04** |
| *-40%* precip. vs. -*20%* precip. | **0.00** | **0.03** |
| *-40%* precip. vs. *0%* change in precip. | 0.13 | 0.18 |
| *-40%* precip .vs. *50%* precip. | **0.00** | **0.00** |
| *-20%* precip. vs. *0%* change in precip. | 0.07 | 0.12 |
| *-20%* precip. vs. 5*0%* precip. | 0.47 | 0.30 |
| *0%* change in precip. vs. *50%* precip. | 0.16 | **0.01** |

**Table 5S.** Average (%± s.e.) plant richness, evenness, relative cover of forbs (%), graminoids (%) and species-specific foliar cover (%) for clipping vs. unclipping under the precipitation gradient. Data are shown for 2017.

|  |  | Precipitation | | | | | | | | | | | | | | | | | | | | |
| --- | --- | --- | --- | --- | --- | --- | --- | --- | --- | --- | --- | --- | --- | --- | --- | --- | --- | --- | --- | --- | --- | --- |
|  |  | **-100%** | | | **-80%** | | | **-60%** | | | **-40%** | | | **-20%** | | | **0% change** | | | **50%** | | |
| Richness | clipped | 19.3 | ± | 1.9 | 19.0 | ± | 3.1 | 22.7 | ± | 2.7 | 22.0 | ± | 1.5 | 20.0 | ± | 1.5 | 19.3 | ± | 1.2 | 23.0 | ± | 2.0 |
| Evenness |  | 0.6 | ± | 0.0 | 0.7 | ± | 0.0 | 0.7 | ± | 0.0 | 0.6 | ± | 0.1 | 0.6 | ± | 0.1 | 0.6 | ± | 0.0 | 0.7 | ± | 0.0 |
| Forbs |  | 0.4 | ± | 0.1 | 0.3 | ± | 0.0 | 0.3 | ± | 0.1 | 0.2 | ± | 0.1 | 0.2 | ± | 0.0 | 0.3 | ± | 0.0 | 0.3 | ± | 0.0 |
| Graminoids |  | 0.4 | ± | 0.2 | 0.5 | ± | 0.0 | 0.5 | ± | 0.1 | 0.6 | ± | 0.1 | 0.6 | ± | 0.1 | 0.6 | ± | 0.0 | 0.5 | ± | 0.0 |
| *Ambrosia psilostachya* |  | 0.1 | ± | 0.1 | 0.2 | ± | 0.0 | 0.1 | ± | 0.1 | 0.0 | ± | 0.0 | 0.1 | ± | 0.0 | 0.1 | ± | 0.1 | 0.0 | ± | 0.0 |
| *Bothriochloa ischaemum* |  | 0.0 | ± | 0.0 | 0.0 | ± | 0.0 | 0.0 | ± | 0.0 | 0.1 | ± | 0.1 | 0.2 | ± | 0.2 | 0.0 | ± | 0.0 | 0.1 | ± | 0.0 |
| *Calylophus serrulatus* |  | 0.1 | ± | 0.0 | 0.0 | ± | 0.0 | 0.1 | ± | 0.0 | 0.1 | ± | 0.0 | 0.0 | ± | 0.0 | 0.0 | ± | 0.0 | 0.1 | ± | 0.1 |
| *Croton monanthogynus* |  | 0.1 | ± | 0.1 | 0.0 | ± | 0.0 | 0.0 | ± | 0.0 | 0.0 | ± | 0.0 | 0.0 | ± | 0.0 | 0.0 | ± | 0.0 | 0.0 | ± | 0.0 |
| *Dalea purpurea* |  | 0.0 | ± | 0.0 | 0.1 | ± | 0.0 | 0.0 | ± | 0.0 | 0.1 | ± | 0.0 | 0.1 | ± | 0.0 | 0.0 | ± | 0.0 | 0.1 | ± | 0.1 |
| *Dichanthelium oligosanthes* |  | 0.0 | ± | 0.0 | 0.0 | ± | 0.0 | 0.0 | ± | 0.0 | 0.0 | ± | 0.0 | 0.0 | ± | 0.0 | 0.0 | ± | 0.0 | 0.0 | ± | 0.0 |
| *Erigeron strigosus* |  | 0.0 | ± | 0.0 | 0.0 | ± | 0.0 | 0.1 | ± | 0.0 | 0.0 | ± | 0.0 | 0.0 | ± | 0.0 | 0.1 | ± | 0.0 | 0.1 | ± | 0.0 |
| *Lespedeza cuneata* |  | 0.0 | ± | 0.0 | 0.0 | ± | 0.0 | 0.0 | ± | 0.0 | 0.0 | ± | 0.0 | 0.0 | ± | 0.0 | 0.1 | ± | 0.0 | 0.0 | ± | 0.0 |
| *Schizachyrium scoparium* (Dominant) |  | 0.4 | ± | 0.1 | 0.3 | ± | 0.1 | 0.4 | ± | 0.1 | 0.5 | ± | 0.1 | 0.4 | ± | 0.1 | 0.4 | ± | 0.1 | 0.4 | ± | 0.1 |
| *Solidago rigida* |  | 0.1 | ± | 0.0 | 0.0 | ± | 0.0 | 0.0 | ± | 0.0 | 0.0 | ± | 0.0 | 0.0 | ± | 0.0 | 0.0 | ± | 0.0 | 0.0 | ± | 0.0 |
| *Sorghastrum nutans* |  | 0.1 | ± | 0.1 | 0.0 | ± | 0.0 | 0.1 | ± | 0.0 | 0.0 | ± | 0.0 | 0.0 | ± | 0.0 | 0.1 | ± | 0.0 | 0.1 | ± | 0.0 |
| *Sporobolus compositus* |  | 0.0 | ± | 0.0 | 0.1 | ± | 0.0 | 0.0 | ± | 0.0 | 0.0 | ± | 0.0 | 0.0 | ± | 0.0 | 0.2 | ± | 0.1 | 0.1 | ± | 0.0 |
| *Symphyotrichum ericoides* |  | 0.0 | ± | 0.0 | 0.1 | ± | 0.0 | 0.1 | ± | 0.0 | 0.0 | ± | 0.0 | 0.0 | ± | 0.0 | 0.0 | ± | 0.0 | 0.1 | ± | 0.0 |
| Richness | unclipped | 14.0 | ± | 0.6 | 17.0 | ± | 0.6 | 16.7 | ± | 0.3 | 20.3 | ± | 2.3 | 19.0 | ± | 2.1 | 22.0 | ± | 1.5 | 17.0 | ± | 0.0 |
| Evenness |  | 0.6 | ± | 0.0 | 0.7 | ± | 0.0 | 0.7 | ± | 0.0 | 0.6 | ± | 0.0 | 0.7 | ± | 0.0 | 0.6 | ± | 0.0 | 0.7 | ± | 0.0 |
| Forbs |  | 0.3 | ± | 0.1 | 0.2 | ± | 0.1 | 0.3 | ± | 0.1 | 0.3 | ± | 0.1 | 0.2 | ± | 0.0 | 0.3 | ± | 0.0 | 0.3 | ± | 0.1 |
| Graminoids |  | 0.6 | ± | 0.1 | 0.6 | ± | 0.1 | 0.6 | ± | 0.1 | 0.5 | ± | 0.1 | 0.5 | ± | 0.1 | 0.6 | ± | 0.1 | 0.6 | ± | 0.1 |
| *Ambrosia psilostachya* |  | 0.1 | ± | 0.0 | 0.1 | ± | 0.0 | 0.1 | ± | 0.1 | 0.1 | ± | 0.0 | 0.1 | ± | 0.0 | 0.1 | ± | 0.1 | 0.1 | ± | 0.0 |
| *Bothriochloa ischaemum* |  | 0.1 | ± | 0.0 | 0.0 | ± | 0.0 | 0.0 | ± | 0.0 | 0.0 | ± | 0.0 | 0.1 | ± | 0.0 | 0.0 | ± | 0.0 | 0.1 | ± | 0.1 |
| *Calylophus serrulatus* |  | 0.1 | ± | 0.1 | 0.0 | ± | 0.0 | 0.1 | ± | 0.0 | 0.1 | ± | 0.0 | 0.0 | ± | 0.0 | 0.1 | ± | 0.0 | 0.1 | ± | 0.0 |
| *Croton monanthogynus* |  | 0.0 | ± | 0.0 | 0.0 | ± | 0.0 | 0.0 | ± | 0.0 | 0.0 | ± | 0.0 | 0.0 | ± | 0.0 | 0.0 | ± | 0.0 | 0.0 | ± | 0.0 |
| *Dalea purpurea* |  | 0.0 | ± | 0.0 | 0.0 | ± | 0.0 | 0.1 | ± | 0.0 | 0.1 | ± | 0.0 | 0.0 | ± | 0.0 | 0.0 | ± | 0.0 | 0.0 | ± | 0.0 |
| *Dichanthelium oligosanthes* |  | 0.1 | ± | 0.0 | 0.0 | ± | 0.0 | 0.1 | ± | 0.0 | 0.0 | ± | 0.0 | 0.0 | ± | 0.0 | 0.0 | ± | 0.0 | 0.0 | ± | 0.0 |
| *Erigeron strigosus* |  | 0.0 | ± | 0.0 | 0.0 | ± | 0.0 | 0.0 | ± | 0.0 | 0.0 | ± | 0.0 | 0.0 | ± | 0.0 | 0.0 | ± | 0.0 | 0.0 | ± | 0.0 |
| *Lespedeza cuneata* |  | 0.1 | ± | 0.0 | 0.1 | ± | 0.1 | 0.0 | ± | 0.0 | 0.0 | ± | 0.0 | 0.0 | ± | 0.0 | 0.1 | ± | 0.0 | 0.2 | ± | 0.0 |
| *Schizachyrium scoparium* (Dominant) |  | 0.5 | ± | 0.1 | 0.3 | ± | 0.0 | 0.4 | ± | 0.1 | 0.5 | ± | 0.1 | 0.3 | ± | 0.1 | 0.4 | ± | 0.1 | 0.3 | ± | 0.1 |
| *Solidago rigida* |  | 0.1 | ± | 0.0 | 0.0 | ± | 0.0 | 0.0 | ± | 0.0 | 0.1 | ± | 0.0 | 0.0 | ± | 0.0 | 0.0 | ± | 0.0 | 0.0 | ± | 0.0 |
| *Sorghastrum nutans* |  | 0.1 | ± | 0.0 | 0.0 | ± | 0.0 | 0.1 | ± | 0.0 | 0.0 | ± | 0.0 | 0.1 | ± | 0.1 | 0.1 | ± | 0.0 | 0.1 | ± | 0.0 |
| *Sporobolus compositus* |  | 0.0 | ± | 0.0 | 0.2 | ± | 0.1 | 0.1 | ± | 0.0 | 0.0 | ± | 0.0 | 0.1 | ± | 0.1 | 0.0 | ± | 0.0 | 0.2 | ± | 0.1 |
| *Symphyotrichum ericoides* |  | 0.1 | ± | 0.0 | 0.1 | ± | 0.0 | 0.1 | ± | 0.0 | 0.0 | ± | 0.0 | 0.0 | ± | 0.0 | 0.1 | ± | 0.0 | 0.0 | ± | 0.0 |

**Table 6S.** Average (%± s.e.) plant richness, evenness, relative cover of forbs (%), graminoids (%) and species-specific foliar cover (%) for clipping vs. unclipping under the precipitation gradient. Data are shown for 2018.

|  |  | Precipitation | | | | | | | | | | | | | | | | | | | | |
| --- | --- | --- | --- | --- | --- | --- | --- | --- | --- | --- | --- | --- | --- | --- | --- | --- | --- | --- | --- | --- | --- | --- |
|  |  | **-100%** | | | **-80%** | | | **-60%** | | | **-40%** | | | **-20%** | | | **0% change** | | | **50%** | | |
| Richness | clipped | 24.3 | ± | 2.6 | 23.7 | ± | 3.0 | 21.7 | ± | 2.2 | 25.0 | ± | 1.7 | 22.0 | ± | 1.0 | 24.7 | ± | 1.3 | 24.0 | ± | 0.6 |
| Evenness |  | 0.7 | ± | 0.0 | 0.8 | ± | 0.0 | 0.7 | ± | 0.0 | 0.7 | ± | 0.1 | 0.7 | ± | 0.0 | 0.7 | ± | 0.0 | 0.8 | ± | 0.0 |
| Forbs |  | 0.3 | ± | 0.0 | 0.3 | ± | 0.1 | 0.3 | ± | 0.0 | 0.4 | ± | 0.1 | 36.5 | ± | 0.0 | 0.3 | ± | 0.0 | 0.4 | ± | 0.0 |
| Graminoids |  | 0.4 | ± | 0.0 | 0.4 | ± | 0.1 | 0.5 | ± | 0.0 | 0.4 | ± | 0.1 | 54.0 | ± | 0.0 | 0.5 | ± | 0.0 | 0.4 | ± | 0.1 |
| *Ambrosia psilostachya* |  | 0.1 | ± | 0.1 | 0.1 | ± | 0.0 | 0.0 | ± | 0.0 | 0.1 | ± | 0.0 | 11.0 | ± | 0.0 | 0.1 | ± | 0.0 | 0.1 | ± | 0.0 |
| *Bothriochloa ischaemum* |  | 0.0 | ± | 0.0 | 0.0 | ± | 0.0 | 0.0 | ± | 0.0 | 0.0 | ± | 0.0 | 0.0 | ± | 0.1 | 0.0 | ± | 0.0 | 0.1 | ± | 0.0 |
| *Calylophus serrulatus* |  | 0.0 | ± | 0.0 | 0.0 | ± | 0.0 | 0.0 | ± | 0.0 | 0.1 | ± | 0.0 | 7.0 | ± | 0.0 | 0.0 | ± | 0.0 | 0.1 | ± | 0.0 |
| *Croton monanthogynus* |  | 0.0 | ± | 0.0 | 0.0 | ± | 0.0 | 0.0 | ± | 0.0 | 0.0 | ± | 0.0 | 2.2 | ± | 0.0 | 0.0 | ± | 0.0 | 0.0 | ± | 0.0 |
| *Dalea purpurea* |  | 0.0 | ± | 0.0 | 0.0 | ± | 0.0 | 0.0 | ± | 0.0 | 0.1 | ± | 0.1 | 11.0 | ± | 0.0 | 0.0 | ± | 0.0 | 0.1 | ± | 0.0 |
| *Dichanthelium oligosanthes* |  | 0.0 | ± | 0.0 | 0.0 | ± | 0.0 | 0.0 | ± | 0.0 | 0.0 | ± | 0.0 | 2.0 | ± | 0.0 | 0.0 | ± | 0.0 | 0.0 | ± | 0.0 |
| *Erigeron strigosus* |  | 0.1 | ± | 0.0 | 0.0 | ± | 0.0 | 0.1 | ± | 0.0 | 0.0 | ± | 0.0 | 6.2 | ± | 0.0 | 0.0 | ± | 0.0 | 0.1 | ± | 0.0 |
| *Lespedeza cuneata* |  | 0.0 | ± | 0.0 | 0.1 | ± | 0.0 | 0.0 | ± | 0.0 | 0.0 | ± | 0.0 | 0.0 | ± | 0.0 | 0.1 | ± | 0.0 | 0.0 | ± | 0.0 |
| *Schizachyrium scoparium* (Dominant) |  | 0.3 | ± | 0.1 | 0.2 | ± | 0.0 | 0.4 | ± | 0.0 | 0.4 | ± | 0.1 | 45.8 | ± | 0.1 | 0.3 | ± | 0.0 | 0.3 | ± | 0.1 |
| *Solidago rigida* |  | 0.1 | ± | 0.0 | 0.0 | ± | 0.0 | 0.0 | ± | 0.0 | 0.1 | ± | 0.1 | 1.0 | ± | 0.0 | 0.1 | ± | 0.0 | 0.1 | ± | 0.0 |
| *Sorghastrum nutans* |  | 0.1 | ± | 0.0 | 0.1 | ± | 0.0 | 0.0 | ± | 0.0 | 0.0 | ± | 0.0 | 1.2 | ± | 0.0 | 0.0 | ± | 0.0 | 0.0 | ± | 0.0 |
| *Sporobolus compositus* |  | 0.0 | ± | 0.0 | 0.1 | ± | 0.0 | 0.0 | ± | 0.0 | 0.0 | ± | 0.0 | 5.0 | ± | 0.0 | 0.0 | ± | 0.0 | 0.0 | ± | 0.0 |
| *Symphyotrichum ericoides* |  | 0.1 | ± | 0.0 | 0.1 | ± | 0.1 | 0.0 | ± | 0.0 | 0.0 | ± | 0.0 | 0.3 | ± | 0.0 | 0.0 | ± | 0.0 | 0.0 | ± | 0.0 |
| Richness | unclipped | 11.3 | ± | 0.7 | 15.0 | ± | 2.3 | 13.7 | ± | 0.3 | 19.7 | ± | 2.8 | 16.3 | ± | 0.3 | 15.3 | ± | 0.9 | 16.3 | ± | 0.3 |
| Evenness |  | 0.8 | ± | 0.0 | 0.8 | ± | 0.0 | 0.8 | ± | 0.0 | 0.7 | ± | 0.0 | 0.7 | ± | 0.1 | 0.8 | ± | 0.0 | 0.8 | ± | 0.0 |
| Forbs |  | 0.5 | ± | 0.1 | 0.3 | ± | 0.1 | 0.4 | ± | 0.0 | 0.3 | ± | 0.0 | 25.3 | ± | 0.1 | 0.3 | ± | 0.0 | 0.3 | ± | 0.1 |
| Graminoids |  | 0.4 | ± | 0.1 | 0.5 | ± | 0.1 | 0.5 | ± | 0.0 | 0.5 | ± | 0.1 | 53.3 | ± | 0.1 | 0.5 | ± | 0.1 | 0.4 | ± | 0.1 |
| *Ambrosia psilostachya* |  | 0.2 | ± | 0.0 | 0.1 | ± | 0.0 | 0.1 | ± | 0.0 | 0.1 | ± | 0.0 | 10.2 | ± | 0.0 | 0.1 | ± | 0.0 | 0.1 | ± | 0.0 |
| *Bothriochloa ischaemum* |  | 0.0 | ± | 0.0 | 0.0 | ± | 0.0 | 0.0 | ± | 0.0 | 0.0 | ± | 0.0 | 0.0 | ± | 0.1 | 0.0 | ± | 0.0 | 0.2 | ± | 0.0 |
| *Calylophus serrulatus* |  | 0.2 | ± | 0.0 | 0.0 | ± | 0.0 | 0.1 | ± | 0.0 | 0.1 | ± | 0.0 | 6.0 | ± | 0.0 | 0.1 | ± | 0.0 | 0.1 | ± | 0.0 |
| *Croton monanthogynus* |  | 0.0 | ± | 0.0 | 0.0 | ± | 0.0 | 0.0 | ± | 0.0 | 0.0 | ± | 0.0 | 1.2 | ± | 0.0 | 0.0 | ± | 0.0 | 0.0 | ± | 0.0 |
| *Dalea purpurea* |  | 0.0 | ± | 0.0 | 0.0 | ± | 0.0 | 0.1 | ± | 0.1 | 0.1 | ± | 0.0 | 2.0 | ± | 0.0 | 0.0 | ± | 0.0 | 0.0 | ± | 0.0 |
| *Dichanthelium oligosanthes* |  | 0.2 | ± | 0.0 | 0.0 | ± | 0.0 | 0.1 | ± | 0.0 | 0.0 | ± | 0.0 | 2.2 | ± | 0.0 | 0.1 | ± | 0.0 | 0.0 | ± | 0.0 |
| *Erigeron strigosus* |  | 0.0 | ± | 0.0 | 0.0 | ± | 0.0 | 0.0 | ± | 0.0 | 0.0 | ± | 0.0 | 1.2 | ± | 0.0 | 0.1 | ± | 0.0 | 0.1 | ± | 0.0 |
| *Lespedeza cuneata* |  | 0.0 | ± | 0.0 | 0.0 | ± | 0.0 | 0.0 | ± | 0.0 | 0.0 | ± | 0.0 | 0.0 | ± | 0.0 | 0.1 | ± | 0.1 | 0.2 | ± | 0.0 |
| *Schizachyrium scoparium* (Dominant) |  | 0.3 | ± | 0.1 | 0.2 | ± | 0.0 | 0.3 | ± | 0.1 | 0.4 | ± | 0.1 | 30.0 | ± | 0.1 | 0.3 | ± | 0.0 | 0.2 | ± | 0.0 |
| *Solidago rigida* |  | 0.1 | ± | 0.0 | 0.0 | ± | 0.0 | 0.1 | ± | 0.0 | 0.0 | ± | 0.0 | 1.0 | ± | 0.0 | 0.0 | ± | 0.0 | 0.0 | ± | 0.0 |
| *Sorghastrum nutans* |  | 0.0 | ± | 0.0 | 0.1 | ± | 0.1 | 0.1 | ± | 0.0 | 0.0 | ± | 0.0 | 11.0 | ± | 0.0 | 0.1 | ± | 0.0 | 0.1 | ± | 0.0 |
| *Sporobolus compositus* |  | 0.1 | ± | 0.0 | 0.2 | ± | 0.1 | 0.1 | ± | 0.0 | 0.0 | ± | 0.0 | 10.2 | ± | 0.0 | 0.1 | ± | 0.0 | 0.1 | ± | 0.0 |
| *Symphyotrichum ericoides* |  | 0.1 | ± | 0.0 | 0.2 | ± | 0.1 | 0.1 | ± | 0.0 | 0.1 | ± | 0.0 | 5.0 | ± | 0.0 | 0.1 | ± | 0.0 | 0.1 | ± | 0.0 |

**Table 7S.** Measures of plant community changes (species gains, losses and turnover) from 2017 to 2018 in each treatment. Shown are means ± s.e.

| **Precipitation** |  | **Species gains** | | | **Species losses** | | | **Species turnover** | | |
| --- | --- | --- | --- | --- | --- | --- | --- | --- | --- | --- |
| -100% |  | 0.29 | ± | 0.05 | 0.18 | ± | 0.00 | 0.47 | ± | 0.05 |
| -80% |  | 0.29 | ± | 0.07 | 0.23 | ± | 0.03 | 0.52 | ± | 0.10 |
| -60% |  | 0.21 | ± | 0.02 | 0.26 | ± | 0.04 | 0.47 | ± | 0.02 |
| -40% |  | 0.25 | ± | 0.01 | 0.16 | ± | 0.04 | 0.41 | ± | 0.04 |
| -20% |  | 0.24 | ± | 0.05 | 0.24 | ± | 0.03 | 0.49 | ± | 0.08 |
| 0% change |  | 0.25 | ± | 0.03 | 0.26 | ± | 0.07 | 0.50 | ± | 0.04 |
| 50% |  | 0.24 | ± | 0.01 | 0.25 | ± | 0.03 | 0.48 | ± | 0.02 |
|  |  |  |  |  |  |  |  |  |  |  |
| **Clipping alone** |  | **Species gains** | | | **Species losses** | | | **Species turnover** | | |
| clipped |  | 0.30 | ± | 0.02 | 0.20 | ± | 0.01 | 0.50 | ± | 0.02 |
| unclipped |  | 0.17 | ± | 0.02 | 0.29 | ± | 0.02 | 0.45 | ± | 0.02 |
|  |  |  |  |  |  |  |  |  |  |  |
| **Precipitation** |  | **Species gains** | | | **Species losses** | | | **Species turnover** | | |
| -100% | clipped | 0.36 | ± | 0.04 | 0.21 | ± | 0.02 | 0.57 | ± | 0.06 |
| -80% |  | 0.36 | ± | 0.06 | 0.20 | ± | 0.02 | 0.56 | ± | 0.08 |
| -60% |  | 0.23 | ± | 0.03 | 0.26 | ± | 0.03 | 0.49 | ± | 0.05 |
| -40% |  | 0.26 | ± | 0.01 | 0.16 | ± | 0.03 | 0.41 | ± | 0.03 |
| -20% |  | 0.27 | ± | 0.04 | 0.19 | ± | 0.03 | 0.47 | ± | 0.05 |
| 0% change |  | 0.36 | ± | 0.04 | 0.19 | ± | 0.03 | 0.55 | ± | 0.02 |
| 50% |  | 0.26 | ± | 0.02 | 0.21 | ± | 0.03 | 0.47 | ± | 0.01 |
|  |  |  |  |  |  |  |  |  |  |  |
| -100% | unclipped | 0.11 | ± | 0.02 | 0.28 | ± | 0.05 | 0.38 | ± | 0.06 |
| -80% |  | 0.22 | ± | 0.06 | 0.32 | ± | 0.08 | 0.54 | ± | 0.09 |
| -60% |  | 0.15 | ± | 0.00 | 0.30 | ± | 0.03 | 0.46 | ± | 0.02 |
| -40% |  | 0.18 | ± | 0.04 | 0.21 | ± | 0.07 | 0.38 | ± | 0.05 |
| -20% |  | 0.19 | ± | 0.07 | 0.30 | ± | 0.03 | 0.48 | ± | 0.09 |
| 0% change |  | 0.11 | ± | 0.02 | 0.37 | ± | 0.07 | 0.48 | ± | 0.06 |
| 50% |  | 0.20 | ± | 0.01 | 0.23 | ± | 0.02 | 0.44 | ± | 0.03 |


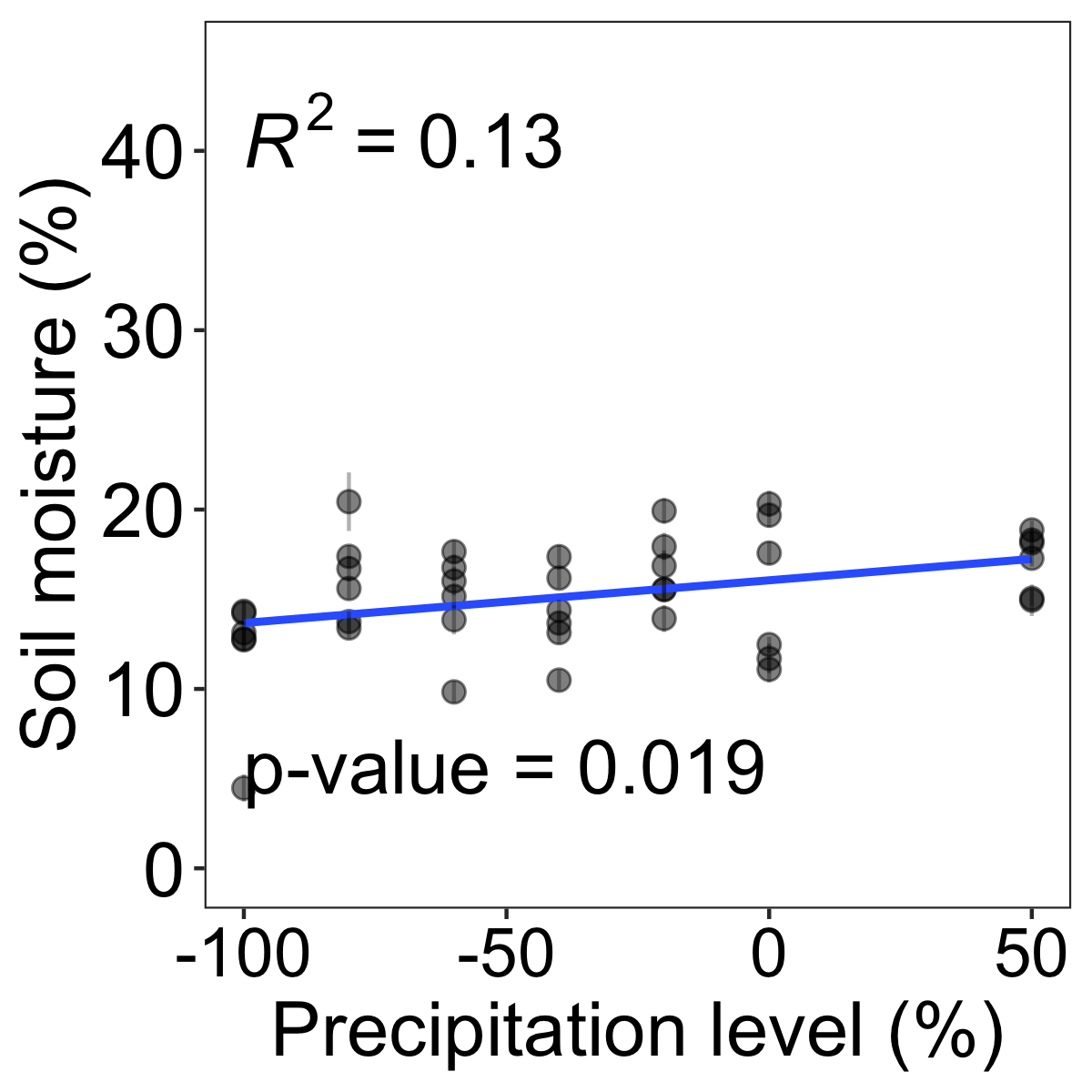

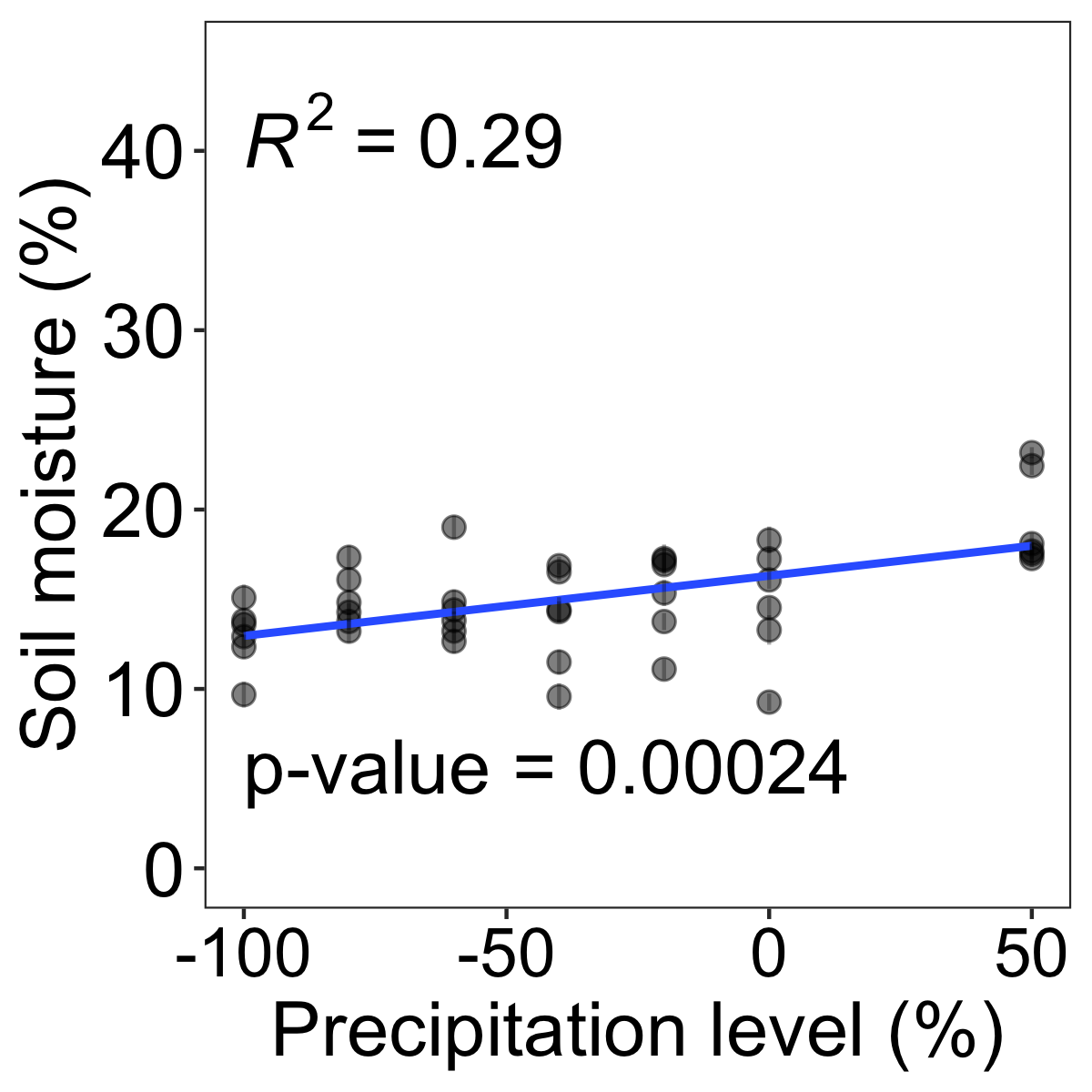


**Figure 1S.** Top panel: Daily soil moisture and precipitation measurements during 2017 and 2018 growing season for all treatments. Bottom panel: Soil moisture content (%, mean±se) regression across the precipitation gradient for each year.

**
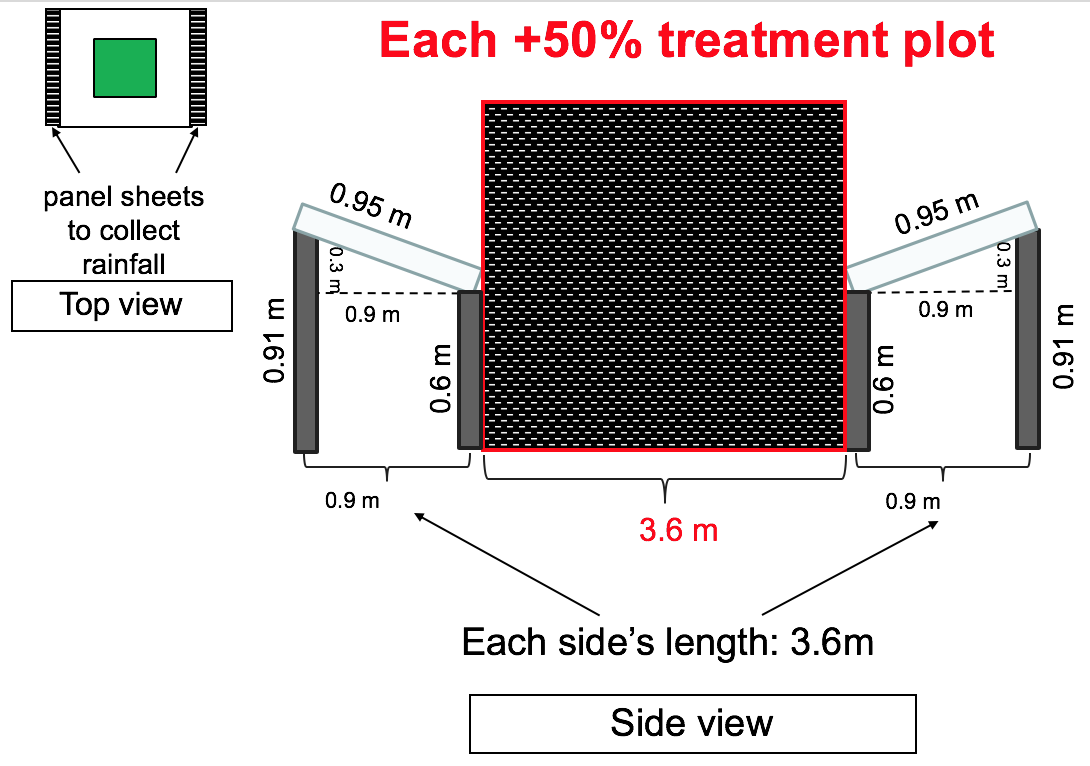
**

**Figure 2S.** Experimental design showing (top panel) arrangement of drought shelters that produced the precipitation gradient distributed in three blocks (n=3, N=21): -100%, -80%, -60%, -40%, -20% precipitation reduction, 0% change (no rainfall change), and 50% precipitation addition; and arrows pointing clipped and unclipped subplots. Top right panel individually illustrates a +50 precipitation addition plot. Bottom panel shows left: illustration of a plot and its subplots, and right: phot of plot at Kessler Atmospheric Ecological Field Station, in Washington, Oklahoma. Subplots were clipped once a year during the growing season (clipping treatment) or left unclipped (unclipping treatment or control).

**Figure 3S.** Average effect sizes (Cohen’s D) and 95% confidence interval (bars) of relative abundance (foliar cover %) for most common species within precipitation treatments. Year 2018 (after treatment application) was compared to year 2016 (before treatment application), including the control treatment (i.e. 0% precipitation). Red circles + bars denote significant effect sizes; red circles + bars to the right indicate positive effect sizes, and red circles + bars to the left indicate negative effect sizes.

**Figure 4S.** Average effect sizes (Cohen’s D) and 95% confidence interval (bars) of relative abundance (foliar cover %) for most common species in clipping treatment and unclipping conditions. Year 2018 (after treatment application) was compared to year 2016 (before treatment application), including the control treatment (i.e. unclipped plots). Red circles + bars denote significant effect sizes; red circles + bars to the right indicate positive effect sizes, and red circles + bars to the left indicate negative effect sizes.

**Figure 5S.** Average effect sizes (Cohen’s D) and 95% confidence interval (bars) of relative abundance (foliar cover %) for most common species within precipitation treatments in clipped and unclipped conditions. Year 2018 (after treatment application) was compared to year 2016 (before treatment application), including the controls treatments (i.e. 0% precipitation and unclipped plots). Red circles + bars denote significant effect sizes; red circles + bars to the right indicate positive effect sizes, and red circles + bars to the left indicate negative effect sizes.

**Figure 6S.** Rank abundance curves for each replicate (different symbols) in each precipitation treatment (-100%, -80%, -60%, -40%, -20%, 0% change[control], +50% precipitation) demonstrate how the community changed in 2017 and 2018. The tail of the rank abundance curve shows rare species. Illustrated are species that were significantly affected by precipitation (*P* < 0.5, Table 1), but also important species in the system, such as the dominant species, *Schizachyrium scoparium* and sub-dominant *Sorghastrum nutans* shown, respectively, in shades of blue and orange. *Erigeron strigosus* (purple shades) increased in relative abundance with more precipitation, while *Croton monanthogynus* (yellow shades) decreased.
