# Supplementary figures and images for: Drought mildly reduces plant dominance in a temperate prairie ecosystem across years
Source: Ecol Evol. 2020 Jun 1;10(13):6702–13. doi: 10.1002/ece3.6400 (PMC7381580; doi:10.1002/ece3.6400)

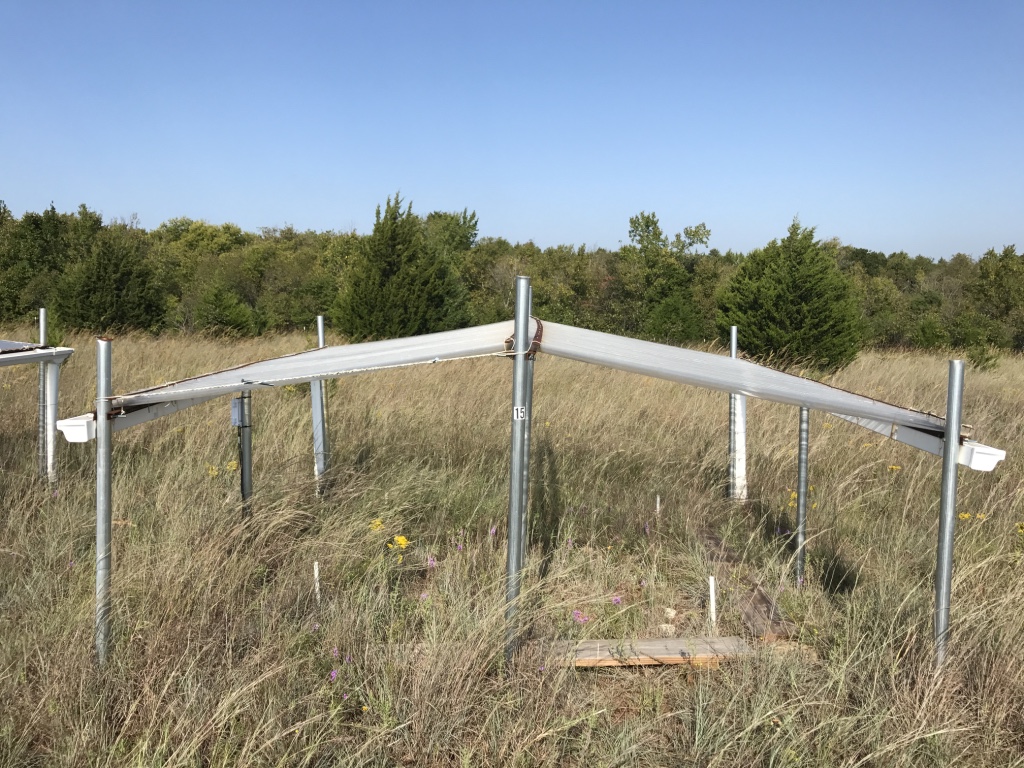

Supplement: Supplementary file 1 — Supplementary Material [file ECE3-10-6702-s001.zip › ece36400-sup-0001-Supinfo/thumb_IMG_5276_1024.jpg]
